# Active and Passive Mineralization of Bio-Gide^®^ Membranes in Rat Calvaria Defects

**DOI:** 10.3390/jfb15030054

**Published:** 2024-02-21

**Authors:** Karol Ali Apaza Alccayhuaman, Patrick Heimel, Stefan Tangl, Stefan Lettner, Carina Kampleitner, Layla Panahipour, Ulrike Kuchler, Reinhard Gruber

**Affiliations:** 1 Department of Oral Biology, University Clinic of Dentistry, Medical University of Vienna, 1090 Vienna, Austria; caroline7_k@hotmail.com (K.A.A.A.); layla.panahipour@meduniwien.ac.at (L.P.); 2 Karl Donath Laboratory for Hard Tissue and Biomaterial Research, University Clinic of Dentistry, Medical University of Vienna, 1090 Vienna, Austria; patrick.heimel@trauma.lbg.ac.at (P.H.); stefan.tangl@meduniwien.ac.at (S.T.); stefan.lettner@meduniwien.ac.at (S.L.); carina.kampleitner@meduniwien.ac.at (C.K.); 3 Austrian Cluster for Tissue Regeneration, 1090 Vienna, Austria; 4 Ludwig Boltzmann Institute for Traumatology, The Research Center in Cooperation with AUVA, 1200 Vienna, Austria; 5 Department of Oral Surgery, University Clinic of Dentistry, Medical University of Vienna, 1090 Vienna, Austria; ulrike.kuchler@meduniwien.ac.at; 6 Department of Periodontology, School of Dental Medicine, University of Bern, 3010 Bern, Switzerland

**Keywords:** collagen membrane, passive mineralization, bone regeneration, animal experiment, histology, micro-CT

## Abstract

Bio-Gide^®^ is a collagen membrane routinely used in guided bone regeneration. Recent studies have shown that this collagen membrane has osteoconductive properties, meaning that it can support the growth of new bone. However, it has also been observed that the collagen membrane has areas of mineralized fibers which can occur spontaneously and independently of osteoblasts. To better understand how this works, we established a model using minced collagen membranes to reduce the active mineralization of intact collagen membranes in favor of passive mineralization. We thus compared the original intact membrane with a minced collagen membrane in a 5 mm calvarial defect model in Sprague Dawley rats. After three weeks of healing, histology and microcomputed tomography (μCT) were performed. Histological analysis confirmed the osteoconductive properties, with new bone growing inside the intact collagen membrane. However, in minced collagen membranes, the osteoconductive properties were restricted to the defect margins. Interestingly, histology revealed large mineralized areas indicating passive mineralization with no signs of bone formation. In the μCT analysis, the intact collagen membranes caused a higher median mineralized volume (1.5 mm^3^) compared with the minced group (0.4 mm^3^), but this lacked significance (*p* = 0.09). The μCT analysis needs to be interpreted carefully, particularly in defects filled with minced membranes, considering that the mineralized tissue may not necessarily be bone but also the result of passive mineralization. Taken together, the findings suggest that Bio-Gide^®^ collagen membranes support bone formation while also exhibiting potential for passive mineralization.

## 1. Introduction

The clinical use of collagen membranes has become a standard in guided bone and tissue regeneration [1,2,3,4]. The overall goal is to shield away the soft tissue from the area of bone regeneration, considering that bone formation should not be disturbed by any invading fast-growing soft tissue [5]. Collagen membranes are also required to provide a biomechanical stable environment as any movements of bone grafts hinder or at least delay the process of bone formation and thus graft consolidation [6]. It is therefore not surprising that collagen membranes, but also synthetic membranes, such as those prepared from non-resorbable polytetrafluoroethylene membrane, are used in contour augmentations, in horizontal and vertical augmentation, and in periodontal regeneration [2,7]. However, from this clinical perspective, the collagen membranes similar to the synthetic membranes are simply a barrier and mechanical support of the augmented area—but recent evidence extends the possible spectrum of activities that possibly support the use of collagen membranes as no longer being only a barrier [5].

Collagen membranes are usually of xenogeneic origin, having been subjected to a sequential series of processing steps that basically remove most of the original cellular components, ending up with a sterile ready-to-use biomaterial. However, the collagen membranes maintain the structural [8,9] and biochemical properties [9,10] of the original tissue. Moreover, the intrinsic biological activity of collagen membranes is reflected by in vitro bioassays, including those that test the activity of the conditioned medium [10], the adsorption of growth factors [11,12], and the cellular response upon seeding [9,13]. In the clinical scenario, however, the collagen membranes are usually moistened with blood from the defect region, where, similar to wound healing, the collagen is inflated by the blood, and the healing cascade is initiated—with neutrophils and macrophages invading the spongy part of the membrane [14]. Later on, blood vessels can sprout into the membrane and inflate the area with new host-derived cells while maintaining the barrier and mechanical properties.

Collagen membranes with the trade name Bio-Gide^®^ (Geistlich Pharma AG, Wolhusen, Switzerland) have been identified to support the growth of new bone [15,16,17], as confirmed by our observations [18,19,20,21,22]. Thus, by definition, the membranes possess osteoconductive properties. We treated rat calvaria defects, where after three weeks, the collagen fibers embedded in the new bone remained visible. Thus, the collagen fibers provided a scaffold or an osteophilic matrix, guiding the bone toward the defect center. Interestingly, there were areas where the collagen fibers appeared mineralized but not obviously due to bone formation by osteoblasts. Thus, these early observations have raised the possibility that apart from their osteoconductive properties, the collagen membranes possess a further characteristic, namely the capacity to support spontaneous but passive mineralization, a process that is termed intrafibrillar mineralization of collagen fibrils. While our previous observations support this hypothesis, further evidence is needed to confirm this claim.

In previous research, we used intact collagen membranes with full integrity, thus allowing bone to penetrate the collagen membranes originating from the defect margins [18,19,20,21,22]. In this setting, it cannot be ruled out that the collagen fibers simply guide the new bone to penetrate the membrane or, if the collagen membrane has an osteoinductive property supporting the osteogenic differentiation of mesenchymal cells, that they, together with blood vessels [23], are sprouting into the membrane. To test this scenario, we implemented a model with minced collagen membranes to interrupt their osteoconductivity but maintain the potential osteoinductive properties. Thus, the model allows us to make observations of what happens in the independent pieces of collagen membranes that become islands in the defect, not necessarily connected to the host bone. This model can also be considered as an attempt to propose minced collagen membranes to serve as a particulated bone graft, at least in contained defects with stable biomechanical conditions.

The results of this research support the previous observations that intact collagen membranes guide new bone growth moving inside the spongy part of the membranes, in addition to the bone growing outside the membrane. With the minced membranes, however, this scenario is restricted to the defect margin. In the center of the defect where the minced collagen membranes appeared as independent islands, occasionally, clear signs of a passive mineralization were observed, supporting the overall hypothesis that Bio-Gide^®^ collagen membranes not only possess osteoconductive properties but also enable intrafibrillar mineralization of collagen fibrils, hence the passive mineralization.

## 2. Materials and Methods

### 2.1. Study Design

The present study was conducted at the Department of Biomedical Research at the Medical University of Vienna, adhering to the ARRIVE guidelines. Approval was obtained from the Medical University of Vienna ethical review board for animal research as well as the Austrian Federal Ministry of Education, Science, and Research (BMBWF-66.009/0399-V/3b/2018). Adult male Sprague Dawley rats weighing between 200 and 300 g were randomly selected for the experiment. They were assigned to receive either an intact collagen membrane (*n* = 10; Bio-Gide^®^, Geistlich, Wolhusen, Switzerland) or a respective minced collagen membrane (*n* = 11). Minced collagen membranes were prepared by cutting squares with a sterile scissor with a length of approximately 1.5–2.0 mm. The rats were housed in a controlled environment with unrestricted access to food and water, following a 12 h dark/light cycle. The allocation of the treatments was concealed from the surgeon until the membrane placement was required on the defect. Throughout analysis, examiners remained blinded to treatment allocation.

### 2.2. Surgery

For the surgical procedure, rats were anesthetized using Ketamine at a dose of 100 mg/kg and xylazine hydrochloride at a dose of 5 mg/kg. A 5 mm defect was created bilaterally in the parietal bone with a trephine drill with a 5 mm outside diameter. Based on the randomized treatment allocations, the defect was then either covered with a collagen membrane or filled with minced collagen membrane. A 6 × 6 mm collagen membrane was inserted in such a way that it overlapped the perimeter of the defect by at least 1 mm at every point, whereas the collagen membrane was minced as indicated and placed into the defect in the other group. Closure of the wounds involved a two-layer technique using resorbable USP 5–0 sutures. To reduce postoperative pain, butorphanol 1.25 mg kg^1^ s.c. and meloxicam 0.15 mg kg1 s.c. were administered. Following a three-week healing period, rats were euthanized by an intracardial overdose of thiopental.

### 2.3. Micro-CT Analysis

The tissue samples underwent fixation using phosphate-buffered formalin (Roti-Histofix 4%, Carl Roth, Karlsruhe, Germany). The micro-CT scans were carried out at 90 kV and 200 A, with an isotropic resolution of 10.3 µm and a 500 ms integration time (Scanco Medical AG, Bruttisellen, Switzerland). Post-acquisition, an open-source image processing program (FIJI, ImageJ, National Institutes of Health, Bethesda, MD, USA) was utilized to orientate the images, aligning the drill direction along the Z-axis and placing the defect approximately at the image center [24]. The ROI was manually delimited and, using a circular cylinder, aligned with the defect’s center and segmented automatically by setting the threshold of 350 mgHA/cm^3^ to distinguish the mineralized tissue from the background. To standardize and streamline the process, we developed an ImageJ ruleset capable of automatically segmenting ROIs from CT images. Subsequently, we quantified parameters such as mineralized volume (MV), mineralized volume fraction per tissue volume (MV/TV), trabecular thickness (Tb.Th), floating mineralization (mm^3^), and defect coverage (%). Floating defines the mineralized areas resembling islands, not connected to the pristine bone.

### 2.4. Histological Analysis

**Figure 1:**
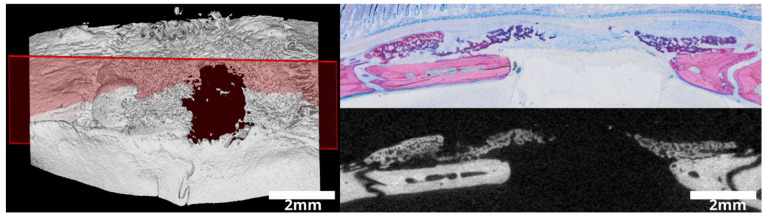
Histology planning in a 3D reconstruction for precise plane placement (the plane in red). The 3D reconstruction depicts a sagittal plane parallel to the midline suture precisely centered within the defect. The image demonstrates the seamless alignment between the µCT slide and the histological slide, indicated by the highlighted red area denoting the position of the plane.

The specimens underwent a dehydration process involving a series of escalating alcohol concentrations. Subsequently, they were embedded in a light-curing resin (Technovit 7200 VLC + BPO; Kulzer & Co., Wehrheim, Germany). Thin-ground sections of all samples were meticulously prepared in a plane parallel to the sagittal suture and through the center of the defect using visualization software (Amira-Avizo 3D 2021.2, Thermo Fisher Scientific, Waltham, MA, USA) (Figure 1). Further processing of the resin blocks was carried out using cutting and grinding equipment (Exakt Apparatebau, Norderstedt, Germany). These sections were stained using Levai–Laczko dye, a combination of azure II and methylene blue, with safranin as a counterstain. The stained slides were systematically scanned and evaluated using an Olympus BX61VS digital virtual microscopy system (DotSlide 2.4; Olympus, Japan, Tokyo) equipped with a 20X objective, providing a resolution of 0.32 µm per pixel. To elucidate and describe additional intricacies, a comprehensive descriptive analysis was performed on the acquired microscopy data.

### 2.5. Statistics

The data collected with the µCT were analyzed using descriptive statistics. We compared the mineralized volume (MV), fraction of mineralized volume per tissue volume (MV/TV), trabecular thickness (Tb.Th), floating mineralization (mm^3^), and defect coverage (%) between the intact and minced groups using a Mann–Whitney U test and displayed them in box plots. R version 4.0.2 was used for analyses and graphing plots [25]. Based on previous research, the sample size was calculated to balance the ability to measure significant differences while reducing the number of animals used.

## 3. Results

### 3.1. Intact and Minced Collagen Membranes: µCT Analysis

**Figure 2:**
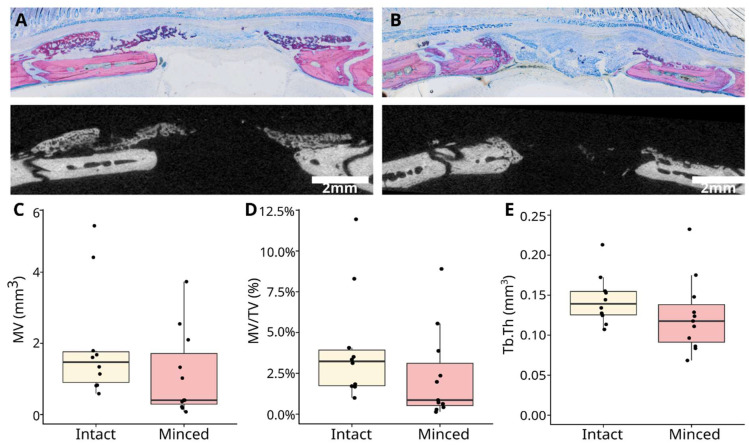
The µCT parameters were assessed in conjunction with histological findings (**A**,**B**). The mineralized volume (MV) within the entire defect exhibited similar measurements, with notable variability among samples when comparing intact collagen membranes with the minced collagen membrane group (**C**). The MV/TV fraction displayed a similar trend (**D**). However, the Tb.Th showed a slightly superior outcome in the intact group compared with the minced group (**E**).

Our previous research showed that the collagen membrane allows an almost complete defect coverage, but other reports from us demonstrate the large variance in this model [18,19,20,21,22]. The data presented in this study echo the variance observed in our previous research. The primary parameters analyzed were the encompassed mineralized volume (MV), the fraction of MV/TV, floating mineralization, and defect coverage (Figure 2). Despite the considerable variability in these parameters, we have a clear trend that is consistent with the findings from the histological analysis. The intact collagen membrane caused a median MV of 1.5 mm^3^ (range 0.9–5.3 mm^3^), while the minced group showed 0.4 mm^3^ (range 0.3–3.7 mm^3^), albeit without achieving statistical significance (*p* = 0.09). Similarly, the MV/TV demonstrated a parallel trend: the intact collagen membranes exhibited a median of 3.2% (range 0.99–11.9%), while the minced membranes had a median of 0.9% (range 0.1–8.9%; *p* = 0.11). It is noteworthy that trabecular parameters, such as trabecular thickness (Tb.Th), were slightly better in the intact group, with a median of 0.14 mm^3^ (range 0.11–0.21 mm^3^), compared with the minced group’s median of 0.12 mm^3^ (range 0.07–0.23 mm^3^), but did not reach statistical significance (*p* = 0.15).

**Figure 3:**
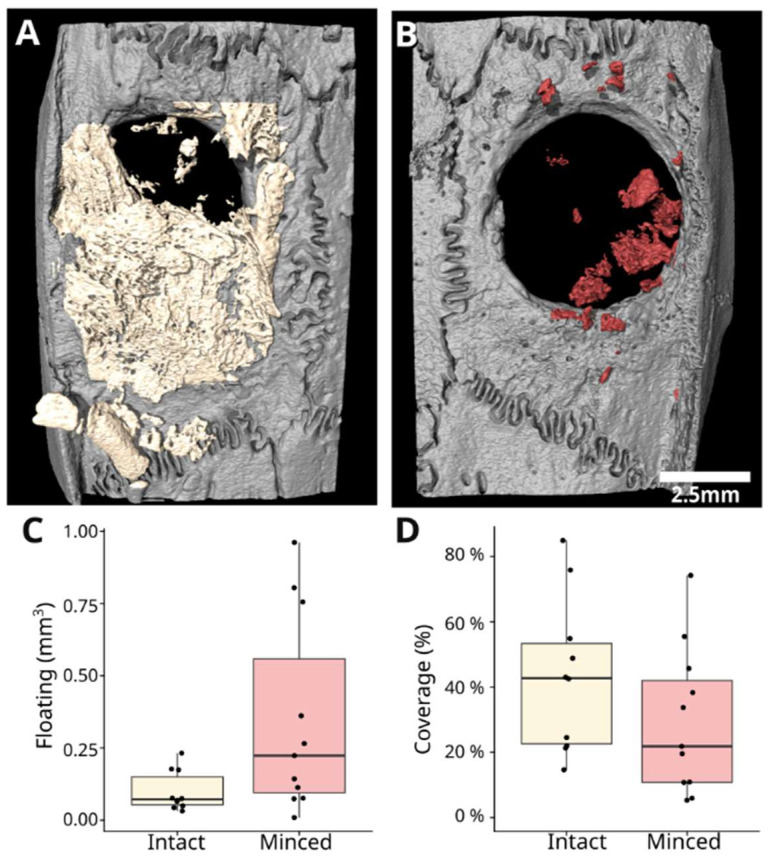
Three-dimensional visualization depicting floating mineralization and defect coverage. Rendered 3D images showcase the distribution of floating mineralization, in which mineralized areas are not connected to the pristine bone, and the extent of defect coverage. In the intact collagen membranes (**A**), a cohesive surface with minimal floating mineralization is observed. Conversely, the minced collagen membranes (**B**) display numerous disconnected “floating” islands. The statistical analysis reveals a significant difference in floating mineralization (*p* = 0.04) (**C**). Additionally, defect coverage appears more robust in the intact group compared with the minced group but did not reach statistical significance (**D**).

Further parameters were analyzed (Figure 3), including floating mineralization, which refers to mineralized areas not obviously connected to the pristine bone. The intact collagen membrane exhibited a median of 0.07 mm^3^ (range 0.03–0.23 mm^3^), while the minced group demonstrated a median of 0.22 mm^3^ (range 0.01–0.96 mm³), indicating statistically significant differences among the groups (*p* = 0.04). Examining defect coverage, the intact collagen membrane displayed a higher median coverage of 42.8% (range 14.6–84.9%), in contrast to the minced membranes with a median of 21.8% (range 5.3–74.2%), though this disparity did not reach statistical significance (*p* = 0.13).

### 3.2. Intact Collagen Membranes: Histological Analysis

**Figure 4:**
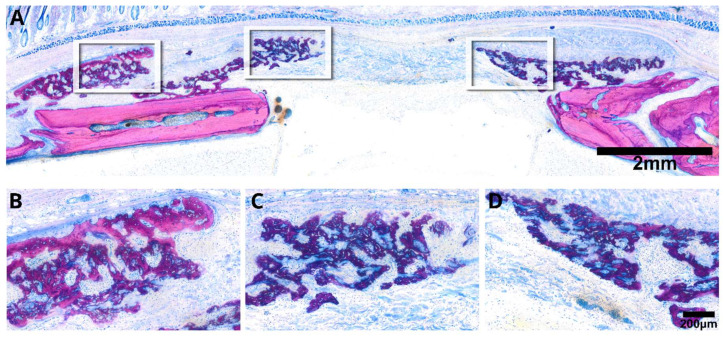
Representative microphotograph depicts the histological characteristics of the intact collagen membrane (**A**). Periosteal new bone growth is evident toward the collagen membrane (**B**) and toward the center (**C**), resembling an attempt to bridge new bone formation from opposing sites (**D**). This hybrid bone exhibits embedded collagen fibers from the membrane within the newly formed bone, showcasing distinctive features (**D**). The intense purple stain is characteristic for less mineralization (**C**,**D**); hence, more dye is taken up by newly mineralized tissue compared with the more mineralized original pristine bone.

**Figure 5:**
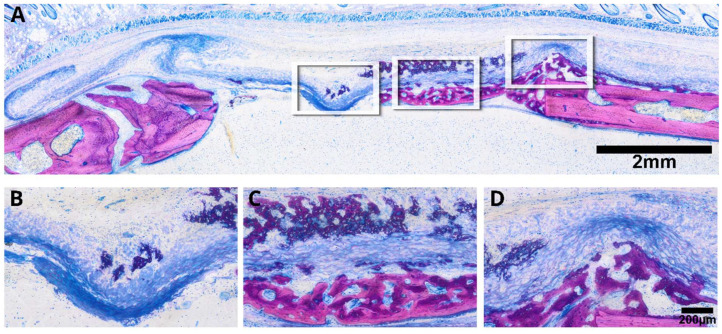
Representative microphotographs depict new bone formation both beneath and above the intact collagen membrane (**A**). Notably, a heavily stained woven bone indicates signs of resorption (**B**). The newly formed bone beneath the membrane displays characteristics of lamellar bone, while above the membrane, a distinctive hybrid bone is observed, intermixed with collagen fibers (**C**). The growth of bone from the edges appears to exert pressure on the collagen membrane, particularly when it is more mature (**D**). Details with arrows marking the locations of the different tissues are in Supplementary Figure S1.

We have recently reported on the osteoconductive properties of membranes, clearly showing the capacity to allow new bone to grow within the spongy areas of the collagen-rich matrix. Moreover, we have observed areas of mineralized collagen fibers where no bone formation was visible, suggesting a passive mineralization independent of osteoblasts [18,19,20,21,22]. The question that arises now is twofold: First, is the collagen membrane simply osteoconductive and thus needs the bone originating from the walls to grow into the defect? Second, does the collagen membrane have potential intrinsic osteoinductive properties which would allow it to initiate osteogenesis at a distance from the pristine bone but injured bone walls? To address the first question, we conducted a histological analysis of selected samples exhibiting substantial mineralization as evidenced by µCT data. Our findings demonstrate that the original collagen membrane effectively guides new bone toward the defect center, portraying a characteristic appearance of immature woven bone with intense staining (Figure 4). Also obvious is the expected new bone formation that originates from the defect margins and growths underneath the collagen membranes, similar to defects left empty. Hence, our investigation affirms the osteoconductive nature of collagen membranes within the calvaria defect model (Figure 5).

### 3.3. Minced Collagen Membranes: Histological Analysis

**Figure 6:**
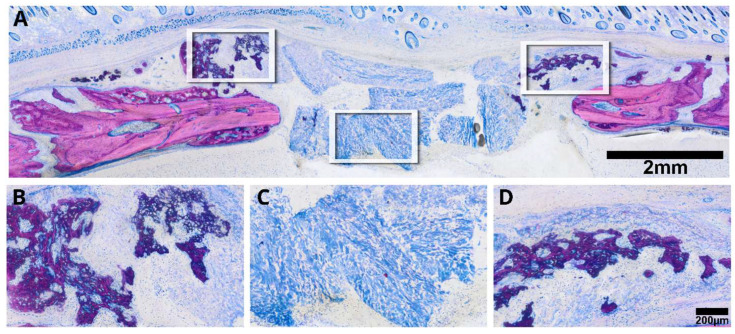
The microphotograph illustrates a minced collagen membrane (**A**), revealing areas of bone formation adjacent to the periosteal bone, exhibiting aspects reminiscent of partially mineralized cartilage (**B**). The collagen membrane pieces resemble floes within the defect (**C**). The newly formed bone exhibits characteristics akin to hybrid bone, as the collagen fibers appear embedded within the newly formed bone structure (**D**). Details with arrows marking the locations of the different tissues are in Supplementary Figure S2.

**Figure 7:**
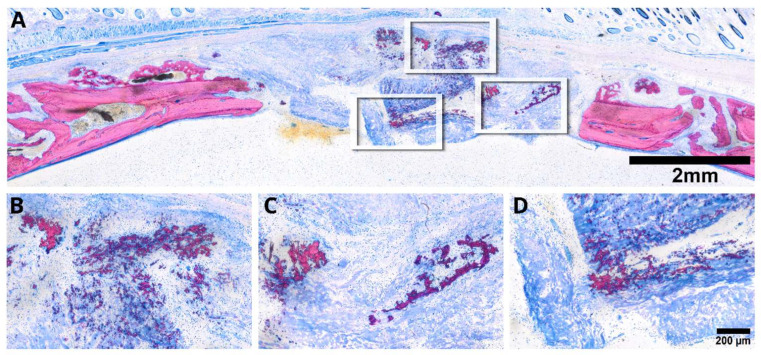
The representative microphotograph (**A**) reveals a minced collagen membrane displaying evident passive mineralization. Clusters of mineralized fibers (**B**) are distributed throughout, resembling bone tissue. However, there is an absence of bone cells around these fibers or an osteoid matrix (**C**). The mineralization of collagen fibrils interconnects the pieces of the collagen membrane (**D**). Details with arrows marking the locations of the different tissues are in Supplementary Figure S3.

To address the second question, we introduced a collagen membrane that was minced prior to its application in a rat calvaria defect. Histological assessments of the specimens selected based on previous µCT analysis distinctly depicted the minced collagen membranes as resembling floes, with around ten pieces dispersed randomly within the defect (Figure 6). Histology revealed areas of osteoconductivity, particularly when the membrane pieces were in immediate proximity to the host bone and its periosteal bone formation. It seems obvious that the new bone growing inside the single membrane pieces originates from the activated periosteum that partially resembles mineralized cartilage, typical for endochondral bone formation. However, in none of the specimens could we identify areas of new bone formation in the center of the defect. These observations clearly suggest that collagen membranes have no intrinsic osteoinductive properties, at least in the rat calvaria defect after a three-week observation period. Of particular interest, and in alignment with earlier research observations [18,19,20,21,22], we identified extensive areas of mineralization within the minced collagen membrane, with no signs of bone formation (Figure 7). The distribution of these mineralized areas appears scattered, lacking a discernible pattern, with some regions connecting to form islands. Moreover, there are signs of resorption visible that provide indirect evidence for osteoclast-like cell activity. These intriguing findings underscore the collagen membrane’s capacity to support passive mineralization.

## 4. Discussion

This research was based on the fundamental questions originating from the classical concept of guided bone regeneration, wherein collagen membranes were initially employed to shield augmented sites undergoing graft consolidation from soft tissue infiltration. These membranes were also tasked with reinforcing the mechanical stability of the augmented site to facilitate the formation of bone rather than fibrous tissue [5]. It was not until recently that the collage membranes were supposed to exceed the passive function and become an active contributor to the overall process of GBR [5]. Support for this claim comes from observations that the collagen membranes used for GBR allow or even support the formation of new bone inside the spongy part of the membranes—at least based on calvaria defect healing models in rats [18,19,20,21,22], dogs [26], and rabbits [27]. This fundamental observation of the osteoconductive properties of collagen membranes has sparked an intriguing hypothesis suggesting that, beyond their role as a barrier, collagen membranes might serve as bone substitutes or even scaffolds. Theoretically, contained defects could be filled with collagen-based granules and scaffolds, leveraging their osteoconductive properties to support bone regeneration [26]. However, our previous research showing the osteoconductive properties of collagen membranes challenged us to predict how a minced collagen membrane behaves in a similar defect. This complexity arises from the fact that new bone growth occurs within the collagen membrane, contrasting with mineralized bone substitutes that solely provide an osteoconductive surface.

We can show here that the intact collagen membranes allow new bone to form inside the spongy part, in addition to the bone growing outside the collagen membrane, and thereby confirm our recent observations [12,18,20,22]. It was obvious that the new bone originates from the periosteum and potentially endosteum of the calvaria; however, it cannot be ruled out that there are also mesenchymal progenitors and thus that new bone originates from the soft tissue and the collagen membranes support its differentiation into bone-forming osteoblasts. To test this hypothesis, we studied the behavior of minced collagen membranes. Interestingly, we noted a lack of spontaneous bone formation in the center of the defects, but occasionally bone formed when minced pieces were in proximity to the periosteum of the calvaria bone. Thus, collagen membranes have no osteoinductive potential, as otherwise the minced pieces would show new bone formation. Our research supports that the bone originates from the host bone and not the soft tissue.

An important observation lies in the distinct mineralization evident in the collagen membrane, particularly when minced, which presents areas with mineralization patterns, distinct from typical bone tissue. While this mineralization remains descriptive, it is likely because of the unique properties of collagen, namely to support the formation of hydroxyapatite when the saturation of calcium and phosphate is reached and crystallization takes place [28,29]. This passive mineralization phenomenon became notably apparent, and was even detectable in the µCT analysis and with electron microscopy [19,20]. This observation further supports our previous research where we identified areas of mineralized collagen fibrils not being embedded on the mineralized matrix produced by the cells, but here we used intact collagen membranes only [18,19,20,21,22]. Together, these findings suggest that passive mineralization is not exclusively observed in collagen membranes but also when membranes are intact. Moreover, it remains unclear if this mineralization is a passive process driven by the oversaturation of calcium and phosphate that accumulate at the defect site and if cells other than osteoblasts possess high levels of alkaline phosphatase activity, forcing the precipitation of the hydroxyapatite on the collagen matrix. On the other hand, however, this observation leads us to a careful interpretation of the µCT data as we cannot distinguish between active osteoblast-derived and passive crystallization-based mineralization. Nonetheless, our finding that minced collagen membranes can undergo spontaneous mineralization, particularly in a rat calvaria defect model, holds a potential clinical implication. First, once the membranes are mineralized, they might become more conductive to bone formation, potentially serving as mineralized bone substitutes. Second, the possibility of inducing ex vivo mineralization in minced collagen membranes opens avenues for creating novel bone substitute materials. This in vitro approach using simulated body fluids is appropriate for surface coating of collagen membranes but not ideal for simulating the in vivo behavior of minced versus intact collagen membranes [30]. In vitro, research based on growing mesenchymal cells on collagen membranes basically shows that the membranes allow osteogenic differentiation, osteoclastogenesis [31], and other behaviors [32], but in vitro models only partially mimic the complex in vivo environment [33].

Further attention needs to be paid to our observation that the cell-derived mineralized tissue produced inside the collagen membranes, most obviously in the minced membranes, is not characteristic of osteoblast-derived bone formation with the typical seams of osteoblasts and the respective osteoid, in which the unmineralized matrix separates the osteoblast from the mineralized bone surface. In contrast, the histological picture shows a form of active mineralization that resembles cells producing a mineralized matrix, more similar to what we know from endochondral bone formation [34]. In this case, the mesenchymal progenitors undergo cartilaginous differentiation and once they reach the status of hypertrophy, they start to produce a mineralized matrix, typically less mineralized than bone and consequently taking up more dye—as observed here with the new matrix staining intensively purple and the bone remaining a pink color [35]. It is, however, likely that mesenchymal cells undergo chondrogenic differentiation, as this is characteristic of areas with unstable mechanical properties, like we know from fracture healing with the callus tissue representing mineralized tissue coming from cartilage cells [6,36]. Chondrogenic differentiation toward hypertrophy became obvious in defects covered with OsseoGuard [15] and to some extent also with the Fibro-Gide membranes [19]. It can be speculated that the calvaria defect model in which defects were bridged by a collagen membrane was exposed to some external forces and also that the oxygen tension on the spongy part of the membrane was perhaps low and favored chondrogenic differentiation. Taken together, we have to be careful stating that the mineralized tissue growing in the spongy part of the collagen membranes originally is bone; this now requires immunophenotyping of markers of chondrogenic differentiation such as collagen types II and X, as well as aggrecan [37], but with the plastic embedded samples, immunostaining is not possible. Hence, future studies should validate our observation using paraffin histology and other techniques to obtain deeper insights into the cellular mechanisms underlying the formation of the cell-derived mineralized tissue.

Our study has several limitations, particularly due to the exploratory nature of our current research. As already mentioned, we seek explanation as to whether the cells forming the mineralized matrix are chondrogenic or osteoblastic in nature. Additionally, further investigations are required to elucidate the mechanisms underlying the passive mineralization of the collagen membranes. We also should mention that, in contrast to our previous research [18,19,20,21,22], we generated two defects that were randomly repeated, which may have led to high variation within the treatment groups. Thus, careful interpretation of the data is essential, even though they support the overall conclusions based on the selective histological samples. Nevertheless, the knowledge gained from the present study can be the basis for future study designs with the clear aim to understand the mechanism of mineralization, the active cellular part, and the passive precipitation part. To some extent, the present research might guide future research trying to implement collagen as granules or scaffolds to support bone generation in general, and in particular in contained defects with stable mechanical conditions.

## 5. Conclusions

Our explorative approach leads us to the suggestion that Bio-Gide^®^ collagen membranes allow biomineralization, independent of bone formation. Moreover, the present research supports previous observations that Bio-Gide^®^ collagen membranes have osteoconductive properties. This research is a primer for future research on the process of biomineralization and how we can take advantage of this phenomenon in the clinic, and on the molecular and cellular mechanisms of how the collagen membrane affects the differentiation of mesenchymal cells toward the osteochondrogenic lineage, as both cell types allow the formation of a mineralized matrix.

## Data Availability

Data sets generated during the current study are available from the corresponding author on reasonable request.

## Supplementary Materials

The following supporting information can be downloaded at https://www.mdpi.com/article/10.3390/jfb15030054/s1: Figure S1: Details with arrows marking the locations of the different tissues; Figure S2: Details with arrows marking the locations of the different tissues; Figure S3: Details with arrows marking the locations of the different tissues.
