# Supplementary material for: Active and Passive Mineralization of Bio-Gide® Membranes in Rat Calvaria Defects
Source: J Funct Biomater. 2024 Feb 21;15(3):54. doi: 10.3390/jfb15030054 (PMC10970795; doi:10.3390/jfb15030054)
Supplement: Supplementary file 1 [file jfb-15-00054-s001.zip › jfb-2811774-supplementary.pdf]

Journal of Functional Biomaterials

Article

Active and Passive Mineralization of Bio-Gide® Membranes in Rat Calvaria Defects

Karol Ali Apaza Alccayhuaman [1,2], Patrick Heimel [2,3,4], Stefan Tangl [2,3], Stefan Lettner [2,3], Carina Kampleitner [2,3,4], Layla Panahipour [1], Ulrike Kuchler [5] and Reinhard Gruber [1,3,6,*]

1 Department of Oral Biology, University Clinic of Dentistry, Medical University of Vienna, 1090 Vienna, Austria; caroline7_k@hotmail.com (K.A.A.A.); layla.panahipour@meduniwien.ac.at (L.P.)
2 Karl Donath Laboratory for Hard Tissue and Biomaterial Research, University Clinic of Dentistry, Medical University of Vienna, 1090 Vienna, Austria; patrick.heimel@trauma.lbg.ac.at (P.H.); stefan.tangl@meduniwien.ac.at (S.T.); stefan.lettner@meduniwien.ac.at (S.L.); carina.kampleitner@meduniwien.ac.at (C.K.)
3 Austrian Cluster for Tissue Regeneration, 1090 Vienna, Austria
4 Ludwig Boltzmann Institute for Traumatology, The Research Center in Cooperation with AUVA, 1200 Vienna, Austria
5 Department of Oral Surgery, University Clinic of Dentistry, Medical University of Vienna, 1090 Vienna, Austria; ulrike.kuchler@meduniwien.ac.at
6 Department of Periodontology, School of Dental Medicine, University of Bern, 3010 Bern, Switzerland
* Correspondence: reinhard.gruber@meduniwien.ac.at

Supplementary Figures

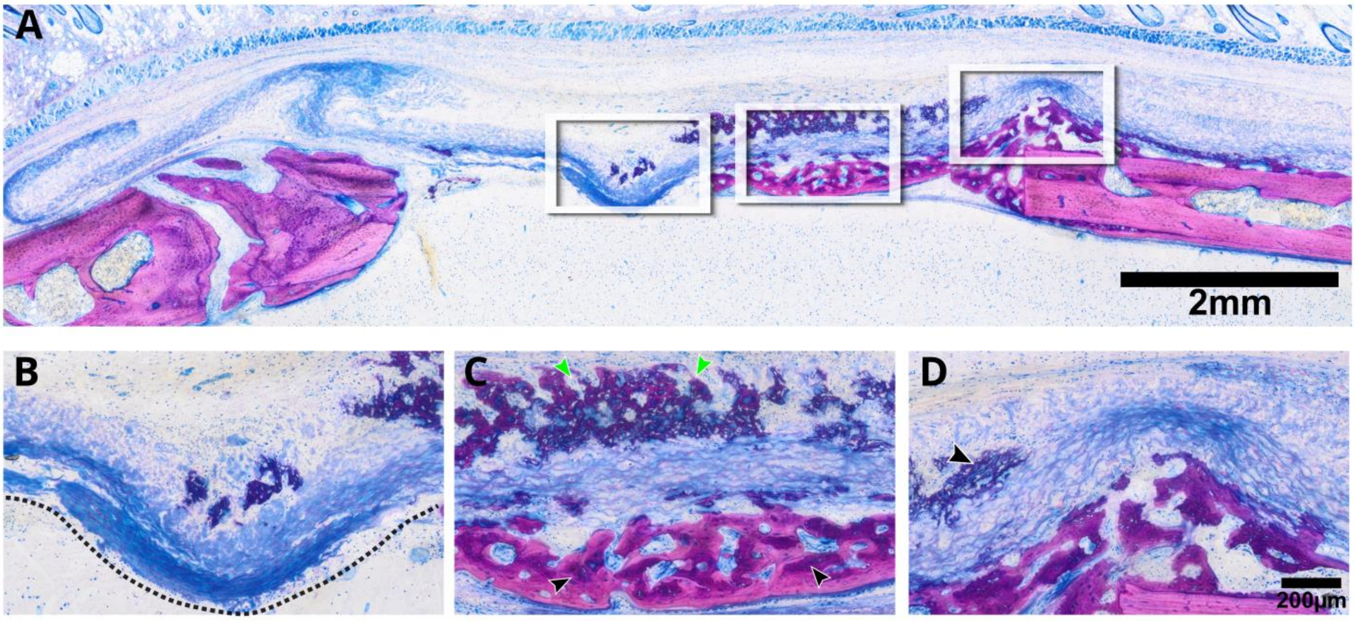

Figure S1. (A) Overview of the intact collagen membrane. (B) Dashed lines delineate the remaining collagen membrane. (C) Green arrows indicate hybrid bone embedded within the collagen membrane, while black arrows mark the lamellar bone beneath the collagen membrane. (D) Isolated cluster of mineralized fibers, highlighted by a black arrow.

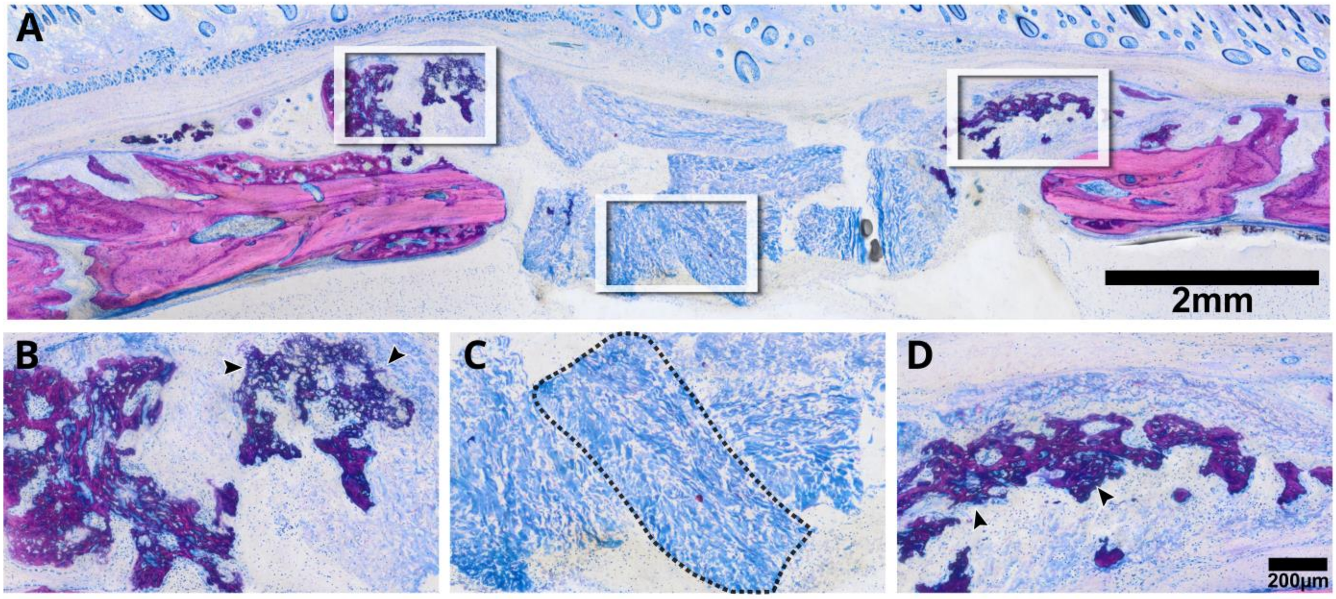

Figure S2 (A) Overview of the minced collagen membrane. (B) Hypertrophic cells, indicative of tissue-like cartilage formation, highlighted by black arrows. (C) Dashed lines demarcate the minced collagen membrane within the defect. (D) Hybrid bone formation within the minced collagen membrane.

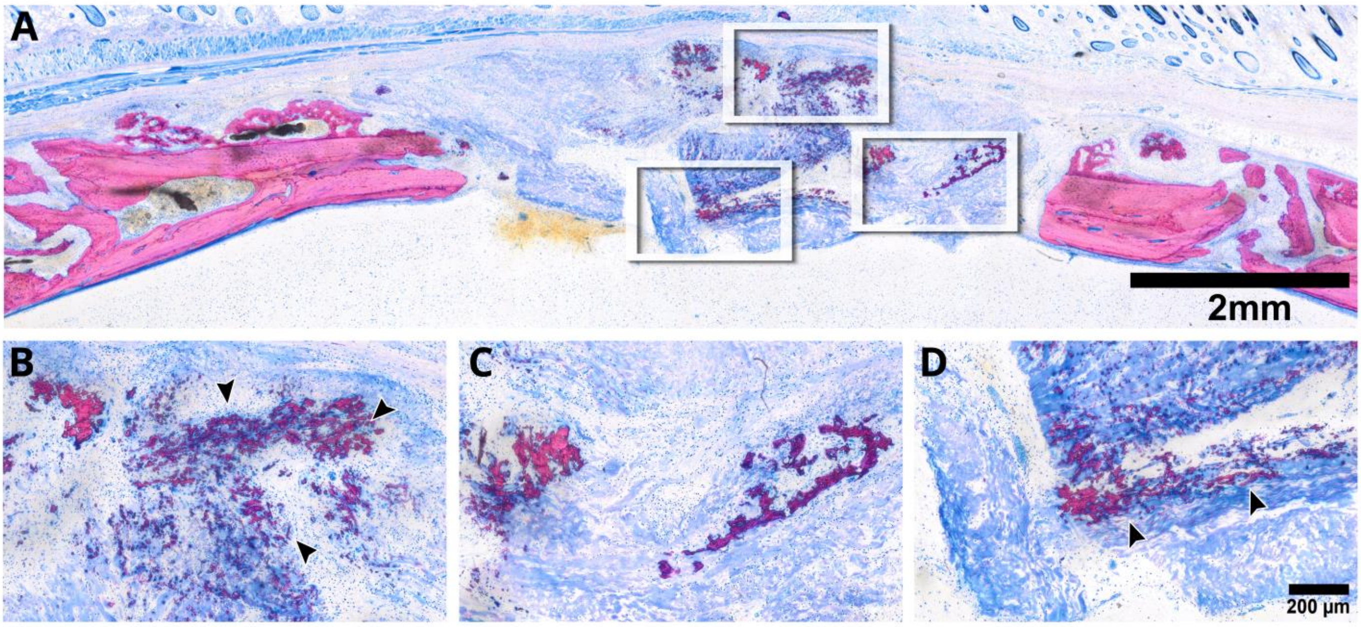

Figure S3. The overview of the minced collagen membrane (A). Mineralized fibers dispersed within the minced membrane are highlighted by black arrows in (B) and (D), forming aggregates that resemble bone in (C).
